# Comparative transcriptomics and comprehensive marker resource development in mulberry

**DOI:** 10.1186/s12864-016-2417-8

**Published:** 2016-02-04

**Authors:** Bushra Saeed, Vinay K. Baranwal, Paramjit Khurana

**Affiliations:** Department of Plant Molecular Biology, University of Delhi South Campus, New Delhi, 110021 India

**Keywords:** *Morus* spp, Single nucleotide polymorphism, Simple sequence repeats, Transcriptome

## Abstract

**Background:**

High potential of *Morus laevigata* and *Morus serrata* has been proposed in the breeding programs for *Morus* sp. However, due to the lack of dense molecular markers this goal is still in its nascent stage and not yet realized. We thus, sequenced the transcriptomes of these two wild *Morus* species and utilized the data for marker development.

**Results:**

We generated 87.0 and 80.3 Mb of transcriptome data from *M. laevigata* and *M. serrata*, respectively. The transcriptomes from *M. laevigata* and *M. serrata*, were assembled into 95,181 and 85,269 transcripts, respectively, and annotated. We identified around 24,049 Simple Sequence Repeats (SSRs), 1,201,326 Single Nucleotide Polymorphisms (SNPs) and 67,875 Insertion-Deletions (InDels). The variants having a higher impact were also identified and their effect was further investigated.

**Conclusions:**

The transcriptome resource from the wildly growing mulberry species developed in this study can find wide applicability in gene identification and/or characterization. It can also contribute immensely in the existing mulberry improvement programs.

**Electronic supplementary material:**

The online version of this article (doi:10.1186/s12864-016-2417-8) contains supplementary material, which is available to authorized users.

## Background

Mulberry plays a crucial role in driving the sericulture industry as it serves as the sole feed for silkworm. In India, four species of mulberry have been reported to occur naturally i.e. *M. indica*, *M. alba*, *M. laevigata* and *M. serrata* [1]*. M. indica* and *M. alba* are cultivated for silkworm rearing whereas the other two grow naturally in the wild. Apart from its uses in sericulture, mulberry is also cultivated for fruit especially *M. laevigata* which produces long sweet fruits, firewood, fodder, and used in furniture, traditional medicine etc. *M. laevigata* grows across the Indian sub-continent and some collections harbor important traits such as disease and termite resistance [2]. *M. serrata* on the other hand, is restricted to higher altitudes (upto 3000 m above sea level) particularly northwestern Himalayas and is known to be tolerant to frost and drought [2, 3]. *M. serrata* also possesses several other important traits such as thicker leaves and higher moisture content and higher moisture retention [1].

Since the ultimate commercial importance of mulberry lies as a feedant for the silkworm, leaf palatability is an important trait directly dependent on leaf water retention capacity, total biomass, and size and weight which are considered significant in the present day breeding programs [4]. Nonetheless, these species possess several agronomically important traits and to utilize the vast genetic potential of these species, hybridization programs between the wild species and cultivated varieties of *Morus* sp. are promising [5, 6].

Additionally, owing to the medicinal and commercial importance of mulberry, a need for developing comprehensive genomic resource has also been felt. In this pursuit, our lab has contributed immensely by generating rich transcriptome-based resources of mature leaf, drought specific transcriptome [7] and root tissue [8] of *M. indica*. The complete chloroplast genome of mulberry was also sequenced [9]. Additionally, these resources have been utilized for generation of Simple Sequence Repeat (SSR) markers for use in mulberry and related species [8, 10]. Recently, though the draft genome of haploid mulberry, *M. notabilis* has been sequenced [11], this is far from complete limiting its practical utility. The growing concern in mulberry is evident from the recent efforts in the expansion of genomic resources [12] and its subsequent utilization in marker development programs [13]. With recent advancements in sequencing technologies, prediction of markers from transcribed regions of the genome has become a method of choice for genotyping particularly for non-model species with less commercial value.

With the above background, in the present study we describe sequencing and generation of large-scale transcriptome based resource for two wild species of mulberry, *M. laevigata* and *M. serrata,* integrated with available information on haploid mulberry *M. notabilis* for DNA based marker development.

## Results and discussion

Even with the advent of next generation sequencing techniques, sequencing whole genomes of ‘less attractive’ or non-model plants/wild species remains impracticable. A fundamental need for introducing wild gene pool in the cultivated varieties of *M. indica* and *M. alba* has been long felt [6]. Thus, two wild species of *Morus* growing in different geographical locations in India were selected for transcriptome sequencing to explore their novel genetic potential and to undertake a comparative analysis. Also, transcriptome sequencing data comprising different tissues (leaf, bark, winter bud, male flower and root) of *M. notabilis* was downloaded from *Morus* genome database (http://morus.swu.edu.cn/morusdb, [14]). Together, these transcriptomes were used for generating a key resource for gene identification, characterization and marker development in the genus *Morus*.

### Transcriptome sequencing using illumina Hiseq2000

Previously, we have reported generation and sequencing of EST libraries of *M. indica* from mature leaves [15], subtractive suppressive hybridization under drought stress [7] and roots [8] of *M. indica*. In the present study, transcriptomes of *M. laevigata* and *M. serrata* were sequenced using Illumina HiSeq2000. A total of 202,465,156 and 195,110,548 reads 2x100 bp in length were generated in *M. laevigata* and *M. serrata,* respectively. Of this, a total of 193,934,604 (95.8 %) and 187,061,908 (95.9 %) paired reads of *M. laevigata* and *M. serrata* respectively were of high quality (Q ≥ 20) and used for further analysis.

### Assembly and transcript clustering

High quality paired reads were used for *de-novo* assembly using Trinity program version r20140413p1 [16] and 182,782 and 163,244 transcripts were identified in *M. laevigata* and *M. serrata,* respectively (Table 1). Clustering of assembled transcripts was undertaken using CD-HIT-EST version 4.6.1 [17]. After clustering the number of transcripts was reduced to nearly half and around 95,181 transcripts were identified with an average length of 908.93 bp in *M. laevigata* (Table 1). Similarly, 85,269 transcripts were identified with an average length of 937.86 bp in *M. serrata* (Table 1). The mapping of HQ reads to the mulberry transcriptomes revealed that around 95.29 and 95.69 % reads mapped to the *M. laevigata* and *M. serrata* unigenes, respectively. The GC content was found to be 41.38 % in *M. laevigata* and 41.43 % in *M. serrata* transcriptomes, respectively*.* Similar results were obtained in a study [18], which reported a GC content of 42.4 % in *Arabidopsis*, 40.9 % in soybean and 40.3 % in chickpea transcripts. However, the GC content of haploid *M. notabilis* genome has been reported to be 35.02 % [11].

**Table 1:** Summary of de-novo assembly statistics of *M. laevigata* and *M. serrata* transcriptomes

	M. laevigata	M. serrata
Number of Transcripts	182,782	163,244
Transcriptome Length (bp)	243,555,783	222,394,131
Average Transcript Length (bp)	1,332.493	1,362.341
N50	2,086	2,140
Number of Clustered Transcripts	95,181	85,269
Clustered Transcriptome Length (bp)	86,513,330	79,970,890
Minimum Transcript Length (bp)	201	201
Maximum Transcript Length (bp)	24,024	14,757
Average Transcript Length (bp)	908.934	937.866
N50	1,612	1,670

**Fig. 1:**
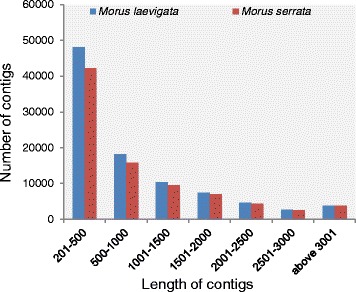
Length distribution of contigs of mulberry transcriptomes

In *M. laevigata,* 48,113 (50.54 %) contigs were found to be in the range of 201–500 bp and 35,940 (37.75 %) contigs in the range of 501–2000 bp (Fig. 1)*.* Similarly in *M. serrata*, around 42,262 (49.56 %) contigs were found in the size range of 201–500 and 32,321 (37.90 %) contigs were found in the range of 501–2000 bp (Fig. 1) suggesting a high probability of finding full-length genes in the present dataset. Orthologous hit ratio has been calculated for both the transcriptomes (*Morus laevigata* and *Morus serrata*). Orthologous hit ratio [19] represents the ratio of length of putative coding region of a unigene divided by the length of the best BLASTx hit considering it as an ortholog. 49,484 unigenes from *Morus laevigata* and 45,260 unigenes from *Morus laevigata* have shown a hit with any sequence in nr database. 11,733 (23.71 %) and 11,412 (25.21 %) unigenes from *Morus laevigata* and *Morus serrata*, respectively, were found to have orthologous hit ratio of >0.9 (Additional file 1: Figure 1). Similarly 22,855 and 21,609 in *Morus laevigata* and *Morus serrata*, respectively, with ortholog hit ratio >0.5.

### Analysis of mulberry transcriptome

**Fig. 2:**
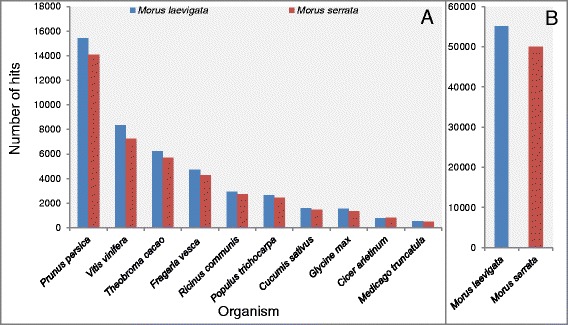
The number of hits obtained from different organisms using BLASTX search against nr database (**a**) number of hits obtained from BLASTX against *M. notabilis* proteome (**b**)

In order to functionally annotate the transcriptomes of *M. laevigata* and *M. serrata,* the transcripts were subjected to BLASTX search against the NCBI nr database and gene ontology (GO) terms were assigned using FastAnnotator (Chang Gung University, Taiwan; [20]). The largest number of sequences showing similarity with *Morus* transcriptomes was with that of *Prunus persica,* followed by *Vitis vinifera* and *Theobroma cacao* (Fig. 2a). Essentially, 51.99 and 53.08 % sequences from *M. laevigata* and *M. serrata*, respectively, showed a hit with any organism in the NCBI nr database (Fig. 2a). The BLASTX against NCBI nr was performed using FastAnnotator and perhaps due the use of an older version of NCBI nr, no hits with *M. notabilis* sequences were obtained, however, a small number of hits from *M. indica* were obtained (data not shown). Nonetheless, we downloaded the proteome of *M. notabilis* with 27,085 sequences from *Morus* genome database (http://morus.swu.edu.cn/morusdb, [14]) and performed BLASTX analysis. A stringent cutoff of 1e-5 e-value was applied to identify the genuine hits. Upon comparison with *M. notabilis* sequences, a significantly higher number of hits i.e. 55,168 (57.96 %) and 50,032 (58.67 %) were obtained in *M. laevigata* and *M. serrata*, respectively (Fig. 2b).

### Gene ontology assignment and analysis

**Fig. 3:**
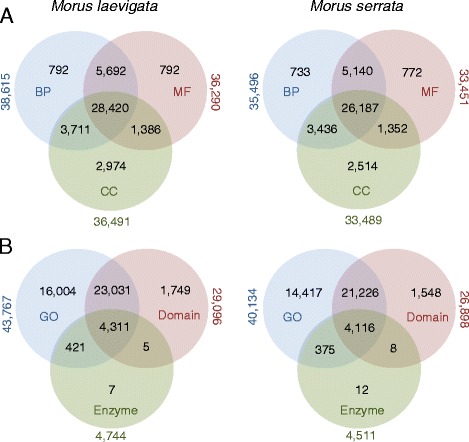
**a** The number of standard GO terms assigned to the transcripts. BP: Biological Process, MF: Molecular Function, CC: Cellular Component. **b** Total GO categories, domains and enzyme classification ids assigned in *M. laevigata* and *M. serrata* unigenes after Blast2GO analysis

The GO ids of unique sequences were categorized into three standard categories: biological process, cellular component and molecular function. Nearly 43,767 and 40,134 unigenes from *M. laevigata* and *M. serrata,* respectively, were assigned to GO terms (Fig. 3a). Around 28,420 and 26,187 unigenes of *M. laevigata* and *M. serrata,* respectively, were assigned all three GO terms (Fig. 3a). Apart from assigning gene ontology ids after BLAST analysis with the NCBI nr database, FastAnnotator also identifies enzyme-encoding transcripts by performing BLAST search against enzyme database and domains based on BLAST against pfam domain database. Thus, a significant number of transcripts from *M. laevigata* (4311; 4.52 %) and *M. serrata* (4116; 4.82 %) were assigned ids in all three types of annotations viz. GO, domains and enzyme (Fig. 3b). Likewise 23,031 transcripts in *M. laevigata* (24.19 %) and 21,226 transcripts in *M. serrata* (24.89 %) were assigned both GO ids and domains (Fig. 3b). The categorization of mulberry unigenes into these three distinct modules can serve as a stepping stone in realizing the full genetic potential of mulberry and further aid in understanding plant gene function in general.

**Fig. 4:**
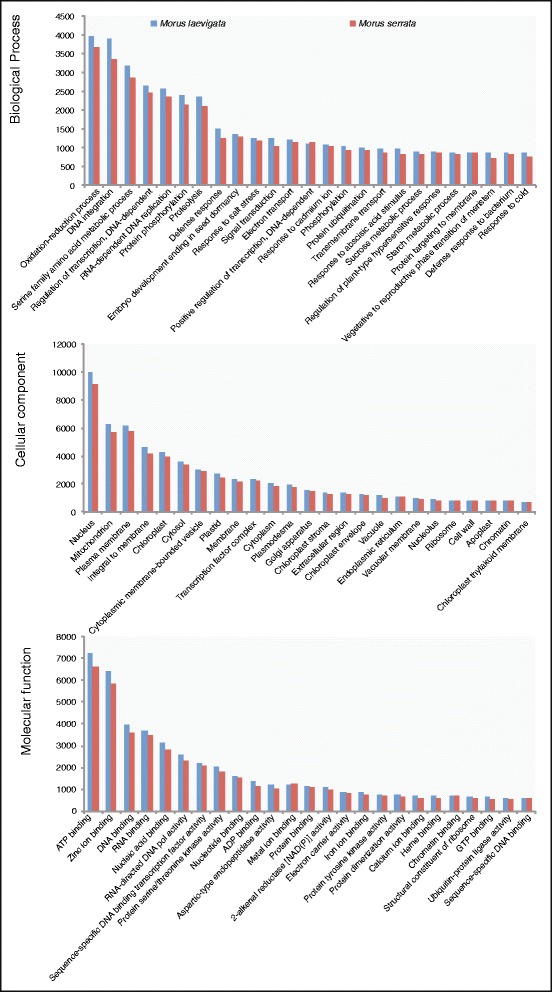
Top twenty-five GO categories represented in *M. laevigata* and *M. serrata* unigenes obtained after Blast2GO against NCBI non-redundant database

Since, the number of GO categories assigned was too large to represent, the results for the top twenty-five categories are presented in Fig. 4. The best-represented groups of biological processes included oxidation-reduction processes, cellular component included nucleus and molecular function included ATP binding and associated processes (Fig. 4). Interestingly, in the top twenty-five categories of biological processes, a high representation of categories involving responses to abiotic stress such as salt, cold and cadmium ion; hormonal response such as ABA stimulus; defense responses and hypersensitive responses; signal transduction and plant microbe interactions was observed (Fig. 4). Amongst the stress related genes, highest number of transcripts was observed in defense response, followed by response to salt stress. The cold responsive genes were also amply represented in the present dataset. This is in broad agreement with the impending use of these two varieties in breeding programs for improving the currently used cultivated varieties as already stated. Among the cellular component category, the highest representation was of nucleus followed by mitochondria and plasma membrane. Furthermore, a high representation of putative regulatory categories such as DNA binding, RNA binding, nucleic acid binding, sequence specific DNA binding, protein binding, chromatin binding and sequence specific DNA binding were among the top twenty five molecular function categories.

### SSR identification in mulberry

SSRs are simple and cost effective markers that can play a key role in improvement of non-model plants such as mulberry. The importance of SSRs in mulberry breeding can be highlighted with the use of SSRs in the development of the first linkage map [21]. Despite their potential advantages there have been a few reports on development of SSR markers in mulberry [8, 10, 22–24]. Recently a database of predicted SSRs from *M. notabilis* has been developed [25]. With the development of high throughput transcriptome based resources, reports of SSR prediction in large datasets have started emerging [26]. Earlier we reported SSR identification in mulberry root ESTs [8] and subsequently in the genic and the non-genic regions [10].

**Fig. 5:**
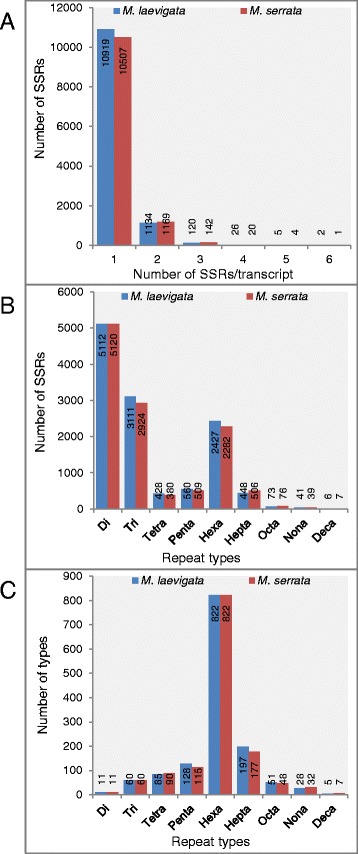
Distribution of total number of SSRs identified in mulberry. **a** Distribution of number of SSRs identified per transcript, **b**. Number of SSRs identified in different repeat units, **c**. Number of types representing different repeat units

In the present study, we identified SSRs from the expressed regions of *M. laevigata* and *M. serrata*. In *M. laevigata* transcriptome, a total of 12,206 SSRs were identified with at least one SSR in 10,919 (11.4 %) transcripts with a density of at least 0.14 SSRs per kb. Similarly, in *M. serrata*, around 11,843 SSRs were identified in 10,507 (12.3 %) with an average of 0.14 SSRs per kb. The maximum number of transcripts showed at least 1 SSR/transcript (Fig. 5a). Most abundantly found SSR types were of di-(41.88 % in *M. laevigata* and 43.23 % in *M. serrata*), tri-(25.48 % in *M. laevigata* and 24.68 % in *M. serrata*) and hexa-nucleotide (19.88 % in *M. laevigata* and 19.26 % in *M. serrata*) repeats (Fig. 5b). In our earlier study, a similar abundance of di-and tri-nucleotide repeats in mulberry transcriptome was observed [8]. The abundance of tri- and hexa- nucleotide repeats does not lead to frameshift that is possibly one of the reasons for its abundance in coding regions [26]. Among the types of repeats, most variability could be seen in the penta-, hexa- and hepta-nucleotide repeats (Fig. 5c). The functional impact of predicted SSR harboring genes was assessed using GO enrichment analysis. The top five significantly enriched categories have been represented in Additional file 2: Figure 2.

### Polymorphism identification

Transcriptome data is extensively used for identification for markers, which being derived from the expressed region of the genome have broad applicability for breeding purposes. Earlier we reported EST based SSRs from root derived transcriptome of *M. indica* [8]. In the present study, a total of 117,052,910 paired reads were obtained from *M. notabilis* out of which 81.01 % reads were aligned with *M. laevigata* de novo assembled transcriptome using Bowtie2 version 2.2.4 [27]. Almost similar alignment rate (80.75 %) was observed when *M. notabilis* was aligned to *M. serrata* transcriptome. Similarly, 97,555,274 paired reads from *M. serrata* were obtained, out of which 93.74 % were aligned to *M. laevigata* de novo assembled transcriptome. The variant calling was done using FreeBayes version 0.9.20-8-gfef284a [28] from the three alignments. Additional file 3: Table 1 summarizes the variants identified initially before filtration.

**Fig. 6:**
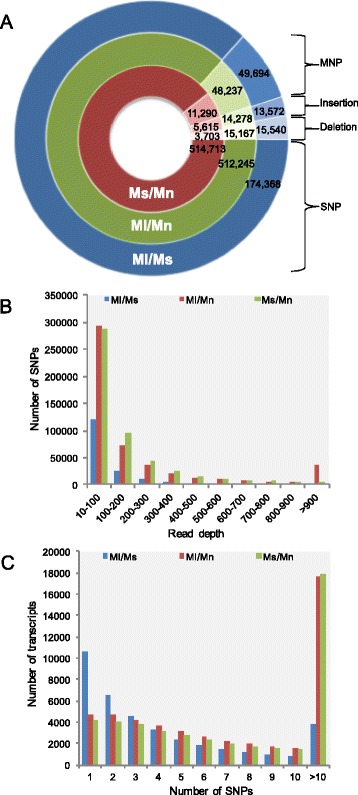
**a** Total number of variants identified in different genotypes after high stringency filtration. *Ml/Mn: M. laevigata/M. notabilis, Ml/Ms: M. laevigata/M. serrata*, *Ms/Mn: M. serrata/M. notabilis,* **b**. Distribution of SNPs with different read depths, **c**. Distribution of number of SNPs identified per transcript. *Ml/Mn: M. laevigata/M. notabilis, Ml/Ms: M. laevigata/M. serrata*, *Ms/Mn: M. serrata/M. notabilis*

The variants were subjected to high stringency filtration using vcftools version 0.1.13 [29]. High quality variants with a quality score above 30, minimum read depth of 10 and 100 % allele balance at heterozygous sites were filtered. We also filtered variants lying in close proximity with each other. Finally, 174,368, 512,245 and 514,713 high quality SNPs were identified with a density of 2.01, 5.92 and 6.43 SNPs/kb (Fig. 6a) in Ml/Ms, Ml/Mn and Ms/Mn, respectively. Apart from SNPs, around 20,608, 77,682 and 78,806 other variants were also identified in Ml/Ms, Ml/Mn and Ms/Mn, respectively, including multiple nucleotide polymorphisms, insertions and deletions (Fig. 6a).

Read depth of least three reads is a reliable quality check for the probabilistic model of SNP prediction. In our dataset, however, the minimum read depth/SNP was in the range of 10–200 reads/SNP that represented the maximum number of SNPs identified in Ml/Ms (86.57 %), Ml/Mn (73.48 %) and Ms/Mn (76.18 %) indicating good quality and high confidence level (Fig. 6b). Correspondingly, around 39.57, 50.81 and 52.59 % transcripts in Ml/Ms, Ml/Mn and Ms/Mn respectively contained at least one SNP. Around 3837, 17,625 and 17,856 transcripts contained more than 10 SNPs in Ml/Ms, Ml/Mn and Ms/Mn, respectively (Fig. 6c). A higher read depth supporting a large number of SNPs when *M. notabilis* transcriptome was used for alignment (possibly because of the higher number of reads present in the initial dataset of *M. notabilis* transcriptome). High read depth per transcript indicates good quality and a higher confidence in predicted SNPs. An average of 4.62, 10.59 and 11.47 SNPs/transcript (in transcripts containing at least one SNP) were identified in Ml/Ms, Ml/Mn and Ms/Mn, respectively. Transitions (A/T to G/C) were found to represent 63.39, 62.66 and 62.60 % of all SNPs in Ml/Ms, Ml/Mn and Ms/Mn, respectively (Additional file 4: Figure 3A). Similarly, the remainder was represented by transversions i.e. 36.60, 37.33 and 37.39 % in Ml/Ms, Ml/Mn and Ms/Mn respectively (Additional file 4: Figure 3A). Nearly similar transition to transversion ratios of 1.73, 1.67 and 1.67 were observed in Ml/Ms, Ml/Mn and Ms/Mn, respectively. The rates of base changes have been represented in Additional file 4: Figure 3B. We also identified around 9318, 29,445 and 29,112 InDels in Ml/Ms, Ml/Mn and Ms/Mn, respectively (Additional file 5: Figure 4). The length of insertions ranged from 1 to 15 bp while the length of deletions ranged from 1 to 17 bp with a density of 10.70, 34.03 and 36.40 InDels/Mb in Ml/Ms, Ml/Mn and Ms/Mn, respectively. Maximum number of InDels was of smaller size ranging from−3 to +4.

### Distribution and effect of variants

**Fig. 7:**
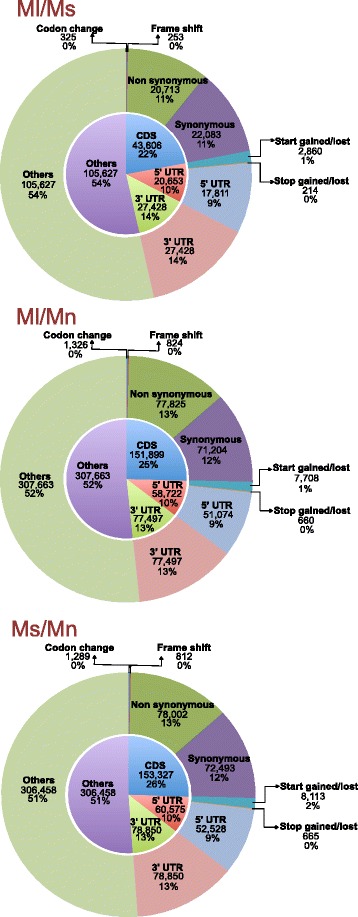
Analysis of the effect of variants on mulberry transcriptomes (number of effects in different regions is shown in inner pie chart, types of effects is shown in outer pie chart). *Ml/Mn: M. laevigata/M. notabilis, Ml/Ms: M. laevigata/M. serrata*, *Ms/Mn: M. serrata/M. notabilis*

SnpEff version 3.0j [30] was employed to understand the overall effect of variants on the transcripts. Around 22−26 % of total polymorphisms were found to be in the coding region. Transdecoder generated GFF file for each assembled unigene transcriptome was used as input for SnpEff. Since, it was derived from transcribed region, a major portion of the variants (50–52 %) could not be annotated (Fig. 7). Nonetheless, around 50 % of total polymorphisms lying in the coding regions of the three genotypes were analyzed. In *M. laevigata*/*M. serrata*, of the 25 % variants in the coding region an equal proportion of non-synonymous and synonymous changes of around 11 % were observed (Fig. 7).

In *M. laevigata*/*M. notabilis*, of the 25 % variants found in the coding region, around 13 % were found to be non-synonymous changes (Fig. 7). Similarly, in *M. serrata*/*M. notabilis*, a higher number of non-synonymous changes (13 %) were observed (Fig. 7). More than 77,000 non-synonymous changes were identified between *M. laevigata* or *M. serrata*/*M. notabilis* indicating a significant evolutionary distance between the two Indian species with *M. notabilis* as expected. A significant number of changes were also observed in the 5’ and 3’ UTR regions suggesting possible changes in these crucial regulatory regions.

### Gene ontology enrichment analysis of high impact variants

**Fig. 8:**
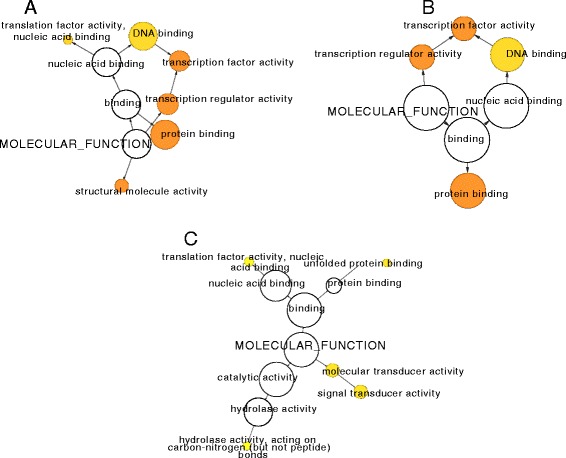
Significantly enriched GO terms in molecular function category. **a** *Ml/Mn: M. laevigata/M. notabilis,* **b**. *Ml/Ms: M. laevigata/M. serrata*, **c**. *Ms/Mn: M. serrata/M. notabilis*

To assess the overrepresentation of GO classes in the annotated variants, gene ontology enrichment analysis was performed using Cytoscape software version 3.0.2 [31] with BiNGO plugin version 3.0.2 [32]. We used high impact variants including InDels, frameshift, non-synonymous coding, stop lost and stop gained for enrichment. The molecular functional classes over represented by variants between Ml/Ms included protein and DNA binding, which might possibly result in differences in protein-protein interactions and interactions with other biomolecules (Fig. 8). Other significantly enriched GO terms included transcription regulator activity and its child term transcription factor activity. Enrichment in this class of GO terms might result in changes between promoter/enhancer or transcription factor binding/activity which might be indicative of overall variability showed by these species in terms of their physiological and biochemical responses. Similarly, in Ml/Mn these categories showed overrepresentation of variants harboring transcripts suggesting major changes in these classes of genes between the genotypes studied. However, in Ms/Mn though the number of significantly enriched categories was much less, still a related category i.e. translation factor activity, nucleic acid binding showed slight enrichment which includes interaction with nucleic acids. Other significantly enriched GO terms included molecular transducer activity and signal transducer activity, which might lead to potential changes in the signal transduction mechanisms. Detection of such genotypic changes can serve as a foundation for understanding of phenotypic difference among different genotypes and breeding.

Interestingly, among the biological processes, an enrichment of SNPs was observed in the response to stimulus category and its children terms i.e. response to abiotic stimulus, biotic stimulus, stress, external and endogenous stimulus (Additional file 6: Figure 5). Enrichment in these categories indicates putative changes that might be responsible for their differential responses. Enrichment in the secondary metabolic processes and related categories such as generation of precursor metabolites and energy, carbohydrate metabolic process and lipid metabolic process was also observed. These changes might be critical as mulberry is a rich source of secondary metabolites with vast medicinal potential [33]. Interestingly, enrichment in developmental process and its children terms was also observed. Given the morphological variation between the two species [5], these categories can serve as a good resource to isolate genes leading to those changes. Intriguingly, upon comparison of *M. serrata* and *M. notabilis*, a higher number of metabolic processes related categories were significantly enriched with variants. This might possibly reflect that these categories may give a substantial contribution to the differences in the two species. In general, significant enrichment was observed between Indian wild species when compared with *M. notabilis* suggesting that the two varieties are closer to each other when compared with *M. notabilis*. The response to biotic stimulus category also showed enrichment, as genes belonging to this category are under rapid evolution to match with the avirulence genes of the pathogens [34, 35]. This information can further be used for identifying/understanding stress responsive mechanisms and changes thereof in different varieties.

### Genotype specific differential expression pattern of genes under various stress conditions

**Fig. 9:**
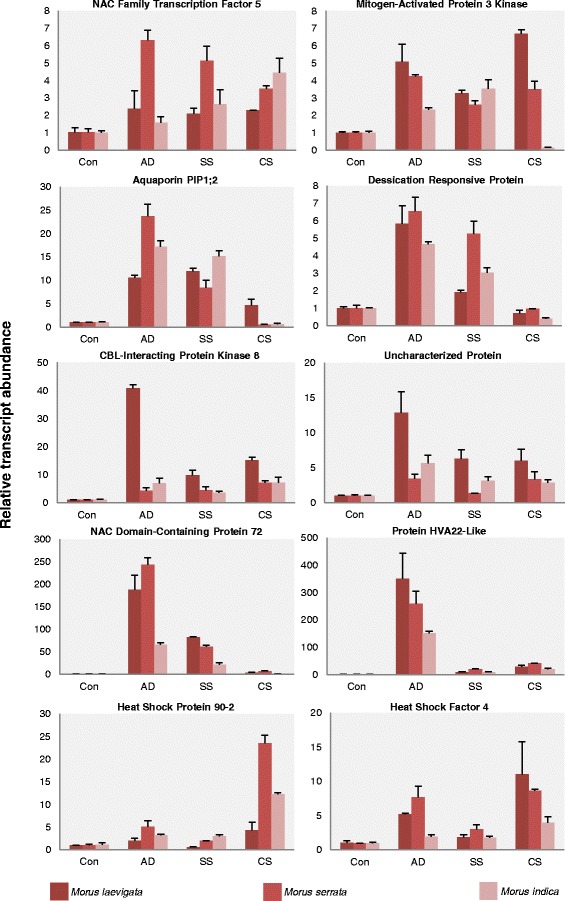
Expression analysis of genes under different environmental cues in *Morus* sp. Con: Mock treated with RO water for five hours, AD: Aerial Drying for five hours, SS: Salt Stress by treatment with 250 mM NaCl for five hours, CS: Cold Stress for five hours. Data represents average of two biological replicates (with three technical replicates each) ± standard error

The expression of several genes identified in both the transcriptomes generated in the present study (*M. laevigata* and *M. serrata*) was analyzed using quantitative real time PCR under altered environmental stimuli (Fig. 9). The expression of these genes was compared with those from *M. indica* cv. K2 (a widely grown cultivar for sericultural practices in India). The involvement of NAC transcription factors in mediating stress responses is well documented [36–38]. Two NAC family genes (NAC family transcription factor 5 and NAC domain containing protein 72) analyzed in the present study showed significantly high up-regulation under simulated dehydration stress (AD) and salt stress (SS) conditions. The gene showing similarity to NAC Domain containing protein 72 in particular showed a very significant up-regulation in dehydration stress in both the tolerant wild species as compared with the susceptible cultivar i.e. *M. indica* cv. K2. Differential expression of rice MAP kinase cascade genes has been reported in response to abiotic stresses [39] likewise, the expression of Mitogen Activated Protein Kinase Kinase Kinase was also found to be up-regulated under dehydration, salt and cold stresses in different mulberry genotypes with significantly higher up-regulation in the wild species. We also analyzed the expression of Plasma membrane intrinsic protein 1;2 (PIP1;2) which showed enhanced expression under dehydration (AD) and salt stress (SS) conditions. The magnitude of induction was the highest in *M. serrata* under dehydration (AD) stress treatment. Interestingly, in *Arabidopsis thaliana*, PIP genes are regulated by drought and ABA [40]. Likewise drought-induced expression of PIP genes has been found to be associated with maintenance of leaf water content [41]. Specifically high expression of dehydration related genes such as HVA22-like, Uncharacterized protein and Dessication responsive protein in dehydration (AD) and salt stress (SS) conditions in wild varieties of mulberry was detected. Overexpression of barley HVA1 gene in mulberry has been implicated in imparting tolerance to salt, drought and cold stresses [42, 43]. The role of CBL-CIPK network is central to modulation of calcium signatures that are translated to different cellular responses [44]. CBL interacting protein kinase 8 (CIPK8) has been shown to play a crucial role in plant responses to nitrate [45]. Maximum induction of expression of CBL interacting protein kinase 8 was observed in aerial drying (AD), salt stress (SS) and cold stress (CS) conditions in *M. laevigata*. Temperature responsiveness of Heat shock proteins [46] and Heat shock factors [47] has also been reported. Thus, we analyzed the expression profile of Heat shock protein 90–2 and Heat shock factor 4 in different mulberry species. Both these genes showed significant up-regulation under cold stress treatment in the wild species with a very strong expression in *M. serrata*.

Modulation of expression and synthesis of a plethora of genes involved in stress signaling are among the most essential adaptive responses of plants to alteration in environmental cues. The expression of several stress related genes in the wild varieties was found to be considerably elevated in both the wild varieties analyzed in the present study. Enhancement in the expression of these genes under drought, salt and cold stresses in the two wild species (*M. laevigata* and *M. serrata*) as compared with the existing cultivar (*M. indica* cv. K2) indicates the potential of these two species in mulberry improvement.

## Conclusions

In the present study, we report generation and functional annotation of a rich transcriptome-based resource for two wild species of mulberry, which have been previously shown to possess important desirable traits such as bigger leaf size, higher leaf moisture retention, and also greater adaptability to adverse conditions. We sequenced and assembled the transcriptome of two *Morus* species i.e. *M. laevigata* and *M. serrata*, and identified around 95,181 and 85,269 unigenes, respectively. The number of unigenes identified is slightly higher than *M. atropurpurea* [13] and *M. multicaulis* [12] possibly due to difference in stages and species selection. The *de-novo* assembled transcriptome was enriched in genes related to abiotic stresses such as salt stress, cold stress and defense responses including hypersensitive responses, signal transduction and plant microbe interactions. These findings corroborate the utility of these two species in mulberry breeding program where genes of desired traits can be introgressed.

In the present study, we also identified SSR and SNP markers. A total of 12,206 and 11,843 SSRs were identified in *M. laevigata* and *M. serrata,* respectively. SNP markers from the three transcriptomes i.e. *M. laevigata*, *M. serrata* and *M. notabilis* were also identified. A total of 174,368, 512,245 and 514,713 high quality SNPs were also identified between Ml/Ms, Ml/Mn and Ms/Mn, respectively. SNP density of 2.01, 5.92 and 6.43 SNPs/kb (with respect to the total transcriptome) was observed which is in range between chickpea [48] and rice [49]. Thus, upon comparison amongst *M. laevigata, M. serrata and M. notabilis* transcriptomes, a rich resource for markers was developed for direct application in mulberry improvement programs. Furthermore, we have identified high impact variant containing transcripts and deduced their ontologies, which could serve as a resource for gene selection for further validation of their role in mediating different responses in these two species. These high impact variants are distributed across important GO terms including transcription, translation, response to stress, anatomical features, and reproductive structure development. This could be suggestive of the differential regulation of these genes amongst these species manifested in the form of their respective phenotypes.

## Methods

### Plant material

For transcriptome sequencing mature leaves of *M. serrata* (Department of Plant Molecular Biology, South Campus, University of Delhi, New Delhi) and *M. laevigata* (Department of Botany, North Campus, University of Delhi, New Delhi) were harvested and flash frozen in liquid nitrogen and stored at−80 °C until further use. Leaves of similar developmental stage (second and third leaves from the apex) were harvested in the month of February.

### RNA isolation and transcriptome sequencing

Total RNA was isolated from mature leaves (approximately 100 mg) of mulberry using modified GITC method [50]. RNA was further purified and DNase treated using Plant mini kit (Qiagen, USA). RNA was quantified using NanoVue (GE Healthcare) and the integrity of the RNA preparation was checked by Agilent 2100 Bioanalyzer® (Agilent Technologies). The library generation was performed using TruSeq RNA Sample Preparation kit and sequenced using Illumina HiSeq2000 with 100 paired end chemistry by commercial service providers (Innovative Life Discoveries, Gurgaon, India) following recommended protocols. Briefly, mRNA enrichment was done using oligo (dT) beads and utilized for cDNA synthesis employing random hexamers followed by the second strand synthesis. The cDNA was fragmented and purified, ligated to adapters and used for sequencing. The two libraries were barcoded and run in a single lane.

### Transcriptome assembly and functional annotation

*De-novo* assembly of mulberry transcriptome was performed using Trinity [16] software version r20140413p1 with default parameters. To generate non-redundant, full-length transcriptome, clustering was done merging contigs with >80 % identity and having a coverage of >80 % to form a single transcript using CD-HIT-EST version 4.6.1 [17]. The high quality (HQ) reads were further mapped to the clustered transcriptome to evaluate the secondary assembly using Bowtie2 version 2.2.4 [27]. In order to functionally annotate the transcriptome resources of mulberry, gene ontology terms were assigned. The sequences were subjected to similarity searches against NCBI nr database using Fast Annotator (Chang Gung University, Taiwan; [20]) using BLASTX program and assigned a putative function based on sequence similarity.

### SSR identification

The identification of simple sequence repeats (SSRs) was done using Perl script obtained from Gramene [51]. The number of repetitive units for different repeats was as follows: Di-9, Tri-6, Tetra-5, Penta-and Hexa-4, rest-3 repeats.

### SNPs and other variants identification

The transcriptomes of *M. notabilis* (leaf, male flower, winter bud, bark and root) were downloaded from *Morus* genome database (http://morus.swu.edu.cn/morusdb, [14]). The downloaded reads from *M. notabilis* were subjected to quality analysis for various parameters including per base sequence quality, sequence length distribution, per base N content and adapter contamination using FastQC version 0.11.2 [52].

For variant identification, the assembled unigenes of *M. laevigata* were used as a reference for aligning reads from *M. serrata* (Ml/Ms) and *M. notabilis* (Ml/Mn) and the unigenes of *M. serrata* were used as a reference for aligning the *M. notabilis* transcriptome (Ms/Mn). The high quality reads were aligned using Bowtie2 version 2.2.4 [27]. FreeBayes version 0.9.20-8-gfef284a [28] was used for calling variants from the three alignments notably Ml/Ms, Ml/Mn and Ms/Mn. The variants were then filtered by vcftools version 0.1.13 [29], using different stringency parameters such as, quality score of at least 30 and above, with a minimum read depth of 10 and variant frequency of 100 %. We also filtered more than two InDels in a 10 bp window and SNPs within three bp of an InDel.

### Evaluation of variants and enrichment analysis

To further our understanding of polymorphism between diverse mulberry species, we evaluated the effect of variants using single nucleotide polymorphism effect predictor SnpEff, version 3.0j (build 2012-09-05) [30]. The variants that could have high impact on transcript/protein (In/Dels, frame shift, stop gained, stop lost and non synonymous coding) were used for enrichment analysis using BiNGO plugin version 3.0.2 [32] of Cytoscape software 3.0.2 [31].

### Stress treatments

For expression analysis, mature leaves of *M. laevigata, M. serrata and M. indica* cv. K2 plants maintained in University of Delhi South Campus (UDSC) were used. For various treatments, detached leaves were immersed in water/solution. Leaves of similar developmental stage (second and third leaves from the apex) were employed for various treatments. Aerial drying (AD): Detached leaves were air dried on a blotting sheet in a culture room maintained at 28 °C for five hours. Cold stress (CS): Detached leaves were placed in RO water in an incubator at a temperature of 6 ± 2 °C for five hours. Control (Con): Detached leaves were mock treated by immersing in Reverse Osmosis (RO) water at 28 °C for five hours. Salt stress (SS): Detached leaves were placed in 250 mM NaCl solution for five hours.

### Quantitative real time PCR

Total RNA was extracted from mulberry leaves following a modified GITC procedure [50] followed by a DNase treatment using RNeasy plant mini kit (Qiagen) according to manufacturers instructions. First strand cDNA was synthesized using 1 μg total RNA in a reaction volume of 25 μl using high capacity cDNA archive kit (Applied Biosystems). The primers were designed by using Primer Express 2.0 software (PE Applied Biosystems) using default parameters and their uniqueness was checked by BLAST tool against nr database (NCBI) and melting curve analysis. Quantitative real time PCR was carried out in Stratagene Mx3005P (Agilent Technologies). The data was normalized using Elongation Factor 1α as an internal control and relative expression values were calculated using ΔΔCT method. The data represents average of two biological replicates with three technical replicates each. List of primers used in this study can be found in Additional file 7: Table 2.

### Data access

The sequence data for the two transcriptomes have been submitted to the Sequence Read Archive (SRA) with the accession numbers SRP068061 for *Morus laevigata* and SRP067869 for *Morus serrata*.

## Additional files

Additional file 1:
**Supporting Figure 1.** Distribution of orthologous hit ratio (A: *Morus laevigata* C: *Morus serrata*). Bar graph showing the frequency of hits at different orthologous hit ratio (B: *Morus laevigata* D: *Morus serrata*) (PDF 8078 kb) Additional file 2:
**Supporting Figure 2.** Significantly enriched top five GO terms (based on *p*-value) of genes harboring SSRs in mulberry transcriptomes (PPTX 68 kb) Additional file 3:
**Supporting Table 1.** Summary of total variants identified in mulberry transcriptomes (PPTX 41 kb) Additional file 4:
**Supporting Figure 3.** Frequency distribution of base changes. Distribution of substitution types i.e. transitions and transversions (A); distribution of base changes (B). Ml/Mn: *M. laevigata/M. notabilis,* Ml/Ms: *M. laevigata/M. serrata*, Ms/Mn: *M. serrata/M. notabilis (PPTX 58 kb)*
Additional file 5:
**Supporting Figure 4.** InDel distribution in mulberry transcriptomes. InDel length distribution in *M. laevigata/M. notabilis* (A); *M. laevigata/M. serrata* (B); *M. serrata/M. notabilis* (C). The x-axis shows InDel length in bp (deletions highlighted with orange line and insertions highlighted by red line) and the y-axis shows the corresponding number of InDels (PPTX 55 kb) Additional file 6:
**Supporting Figure 5.** Significantly enriched GO terms in biological process category in mulberry transcriptomes. Ml/Mn: *M. laevigata/M. notabilis,* Ml/Ms: *M. laevigata/M. serrata*, Ms/Mn: *M. serrata/M. notabilis (EPS 2556 kb)*
Additional file 7:
**Supporting Table 2.** List of primers used for Quantitative Real Time PCR (PPTX 53 kb)
