# Supplementary figures and images for: Comparative transcriptomics and comprehensive marker resource development in mulberry
Source: BMC Genomics. 2016 Feb 4;17:98. doi: 10.1186/s12864-016-2417-8 (PMC4743097; doi:10.1186/s12864-016-2417-8)

A.

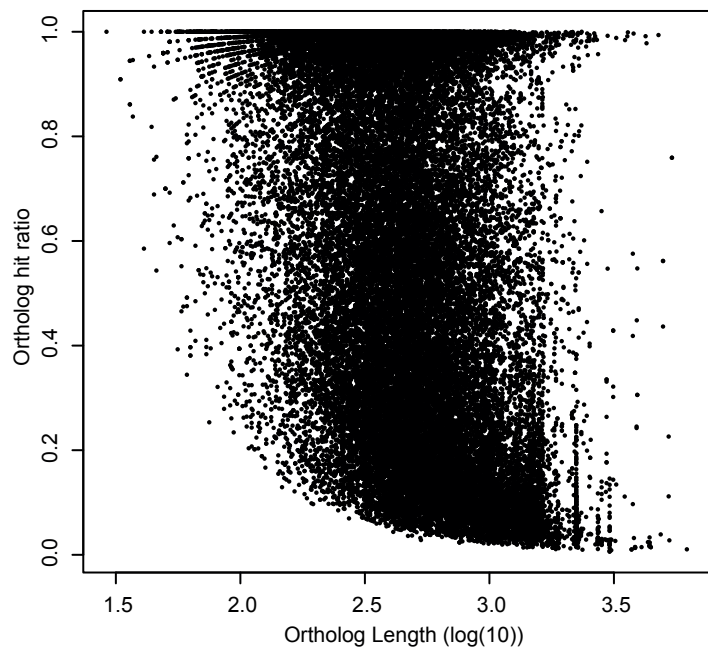

B.

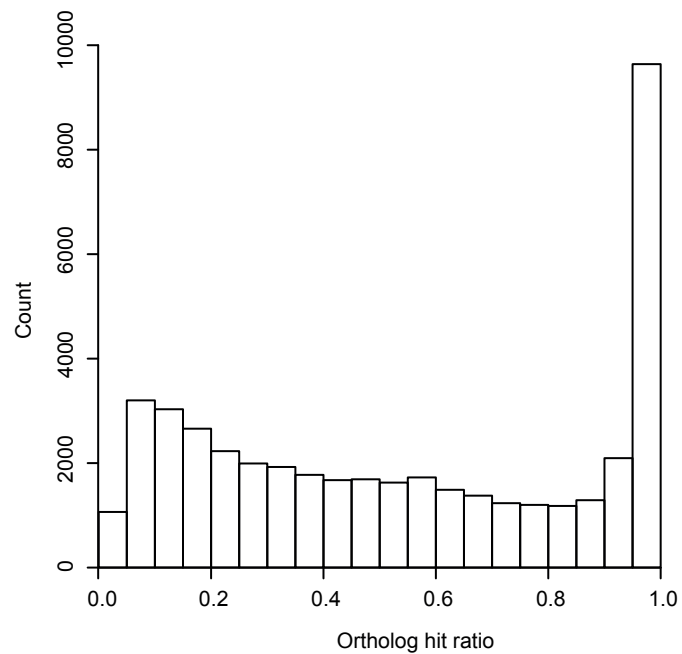

C.

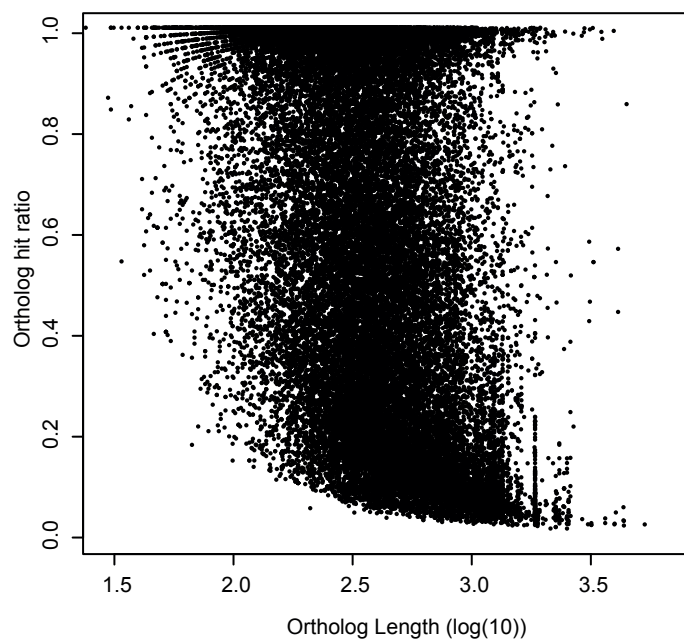

D.

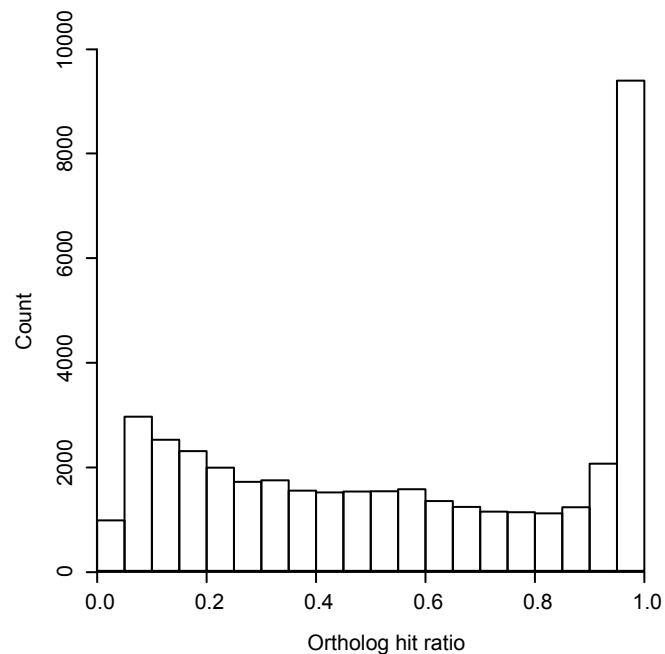

Supporting Figure 1.

Supplement: Additional file 1: — Supporting Figure 1. Distribution of orthologous hit ratio (A: Morus laevigata C: Morus serrata). Bar graph showing the frequency of hits at different orthologous hit ratio (B: Morus laevigata D: Morus serrata) (PDF 8078 kb) [file 12864_2016_2417_MOESM1_ESM.pdf]
