# Supplementary material for: Comparative transcriptomics and comprehensive marker resource development in mulberry
Source: BMC Genomics. 2016 Feb 4;17:98. doi: 10.1186/s12864-016-2417-8 (PMC4743097; doi:10.1186/s12864-016-2417-8)
Supplement: Additional file 2: — Supporting Figure 2. Significantly enriched top five GO terms (based on p-value) of genes harboring SSRs in mulberry transcriptomes (PPTX 68 kb) [file 12864_2016_2417_MOESM2_ESM.pptx]

## Slide 1
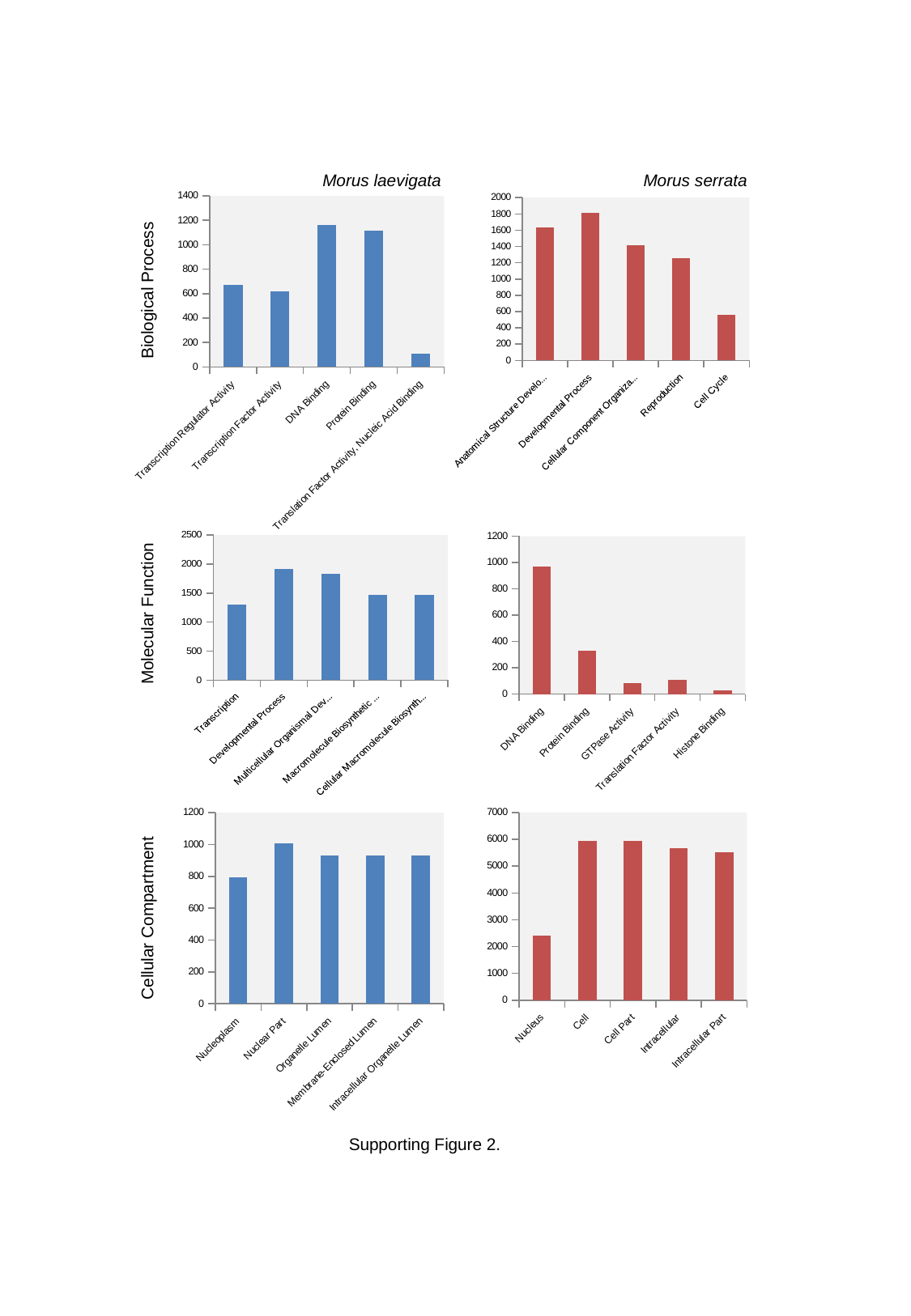

Morus serrata
Morus laevigata
### Chart
| Category | |
|---|---|
| Transcription Regulator Activity | 669.0 |
| Transcription Factor Activity | 621.0 |
| DNA Binding | 1164.0 |
| Protein Binding | 1118.0 |
| Translation Factor Activity, Nucleic Acid Binding | 109.0 |
### Chart
| Category | |
|---|---|
| Anatomical Structure Development | 1638.0 |
| Developmental Process | 1814.0 |
| Cellular Component Organization | 1419.0 |
| Reproduction | 1260.0 |
| Cell Cycle | 564.0 |Biological Process
### Chart
| Category | |
|---|---|
| Transcription | 1301.0 |
| Developmental Process | 1913.0 |
| Multicellular Organismal Development | 1828.0 |
| Macromolecule Biosynthetic Process | 1465.0 |
| Cellular Macromolecule Biosynthetic Process | 1465.0 |
### Chart
| Category | |
|---|---|
| DNA Binding | 967.0 |
| Protein Binding | 329.0 |
| GTPase Activity | 84.0 |
| Translation Factor Activity | 108.0 |
| Histone Binding | 28.0 |Molecular Function
### Chart
| Category | |
|---|---|
| Nucleus | 2410.0 |
| Cell | 5952.0 |
| Cell Part | 5939.0 |
| Intracellular | 5665.0 |
| Intracellular Part | 5505.0 |
### Chart
| Category | |
|---|---|
| Nucleoplasm | 794.0 |
| Nuclear Part | 1007.0 |
| Organelle Lumen | 931.0 |
| Membrane-Enclosed Lumen | 931.0 |
| Intracellular Organelle Lumen | 931.0 |Cellular Compartment
Supporting Figure 2.
