# Supplementary material for: Comparative transcriptomics and comprehensive marker resource development in mulberry
Source: BMC Genomics. 2016 Feb 4;17:98. doi: 10.1186/s12864-016-2417-8 (PMC4743097; doi:10.1186/s12864-016-2417-8)
Supplement: Additional file 3: — Supporting Table 1. Summary of total variants identified in mulberry transcriptomes (PPTX 41 kb) [file 12864_2016_2417_MOESM3_ESM.pptx]

## Slide 1
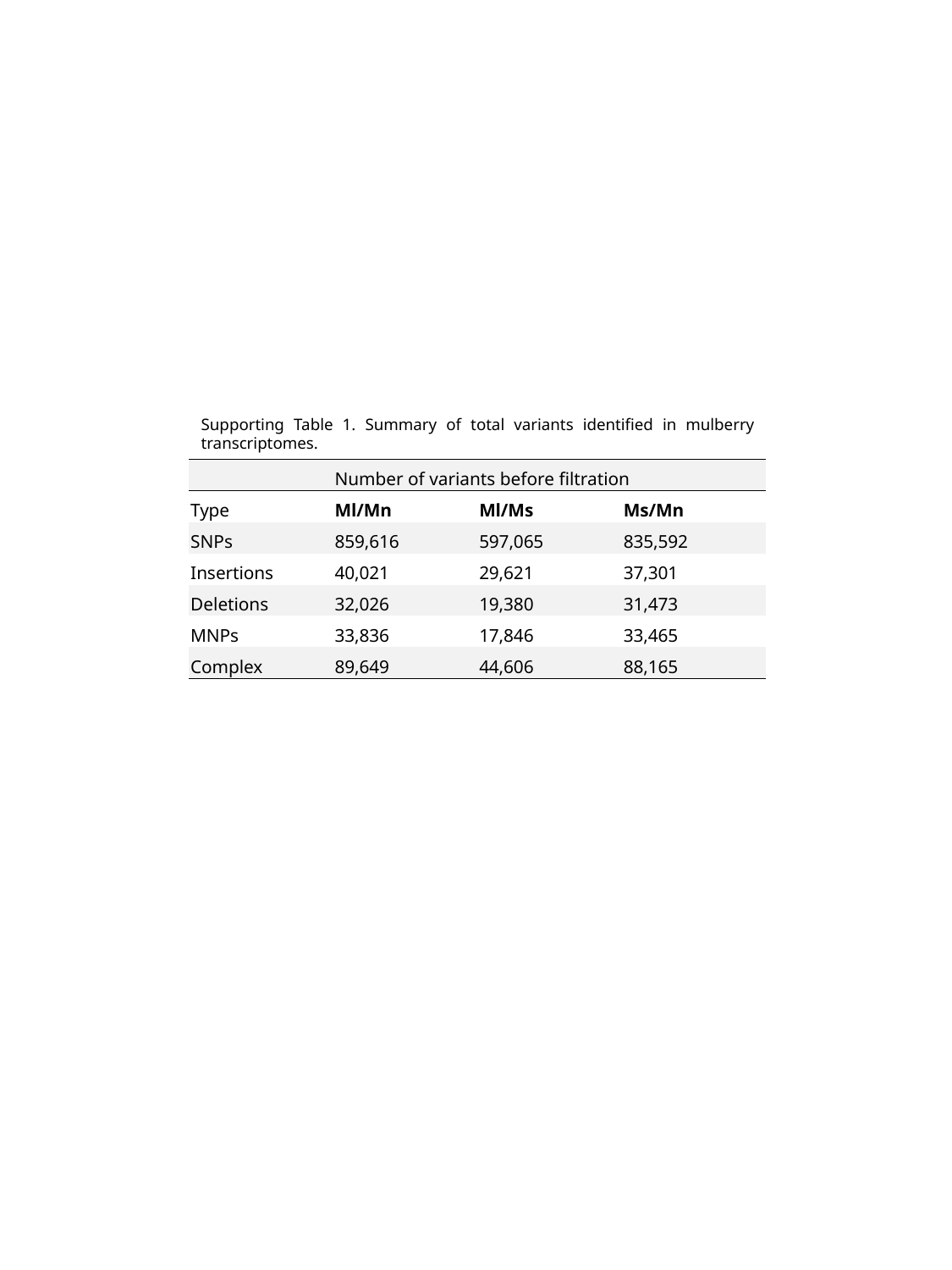

Supporting Table 1. Summary of total variants identified in mulberry transcriptomes.
| | Number of variants before filtration | | |
| --- | --- | --- | --- |
| Type | Ml/Mn | Ml/Ms | Ms/Mn |
| SNPs | 859,616 | 597,065 | 835,592 |
| Insertions | 40,021 | 29,621 | 37,301 |
| Deletions | 32,026 | 19,380 | 31,473 |
| MNPs | 33,836 | 17,846 | 33,465 |
| Complex | 89,649 | 44,606 | 88,165 |
