# Supplementary material for: Comparative transcriptomics and comprehensive marker resource development in mulberry
Source: BMC Genomics. 2016 Feb 4;17:98. doi: 10.1186/s12864-016-2417-8 (PMC4743097; doi:10.1186/s12864-016-2417-8)
Supplement: Additional file 4: — Supporting Figure 3. Frequency distribution of base changes. Distribution of substitution types i.e. transitions and transversions (A); distribution of base changes (B). Ml/Mn: M. laevigata/M. notabilis, Ml/Ms: M. laevigata/M. serrata, Ms/Mn: M. serrata/M. notabilis (PPTX 58 kb) [file 12864_2016_2417_MOESM4_ESM.pptx]

## Slide 1
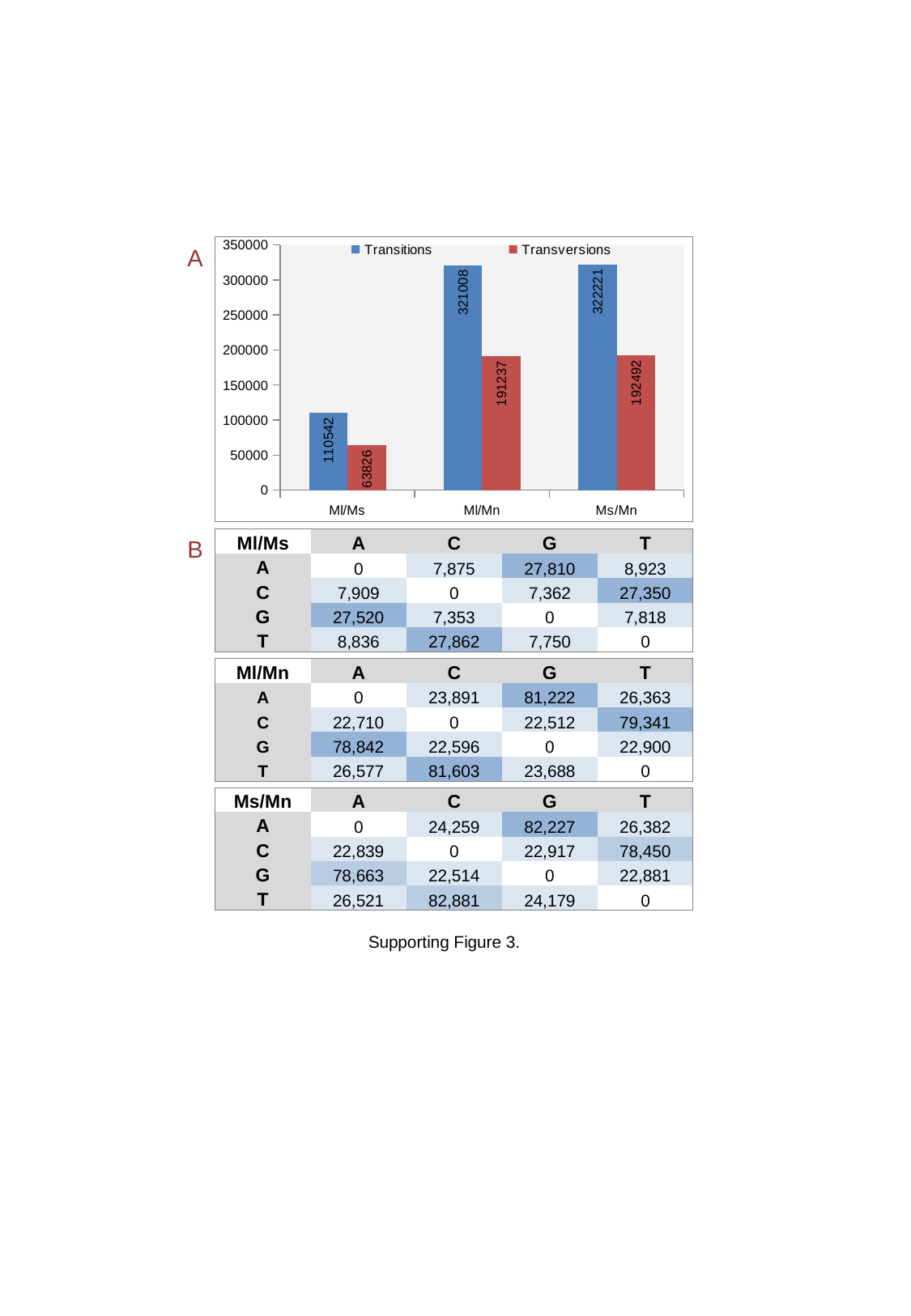

A
### Chart
| Category | Transitions | Transversions |
|---|---|---|
| Ml/Ms | 110542.0 | 63826.0 |
| Ml/Mn | 321008.0 | 191237.0 |
| Ms/Mn | 322221.0 | 192492.0 |B
| Ml/Ms | A | C | G | T |
| --- | --- | --- | --- | --- |
| A | 0 | 7,875 | 27,810 | 8,923 |
| C | 7,909 | 0 | 7,362 | 27,350 |
| G | 27,520 | 7,353 | 0 | 7,818 |
| T | 8,836 | 27,862 | 7,750 | 0 |
| Ml/Mn | A | C | G | T |
| --- | --- | --- | --- | --- |
| A | 0 | 23,891 | 81,222 | 26,363 |
| C | 22,710 | 0 | 22,512 | 79,341 |
| G | 78,842 | 22,596 | 0 | 22,900 |
| T | 26,577 | 81,603 | 23,688 | 0 |
| Ms/Mn | A | C | G | T |
| --- | --- | --- | --- | --- |
| A | 0 | 24,259 | 82,227 | 26,382 |
| C | 22,839 | 0 | 22,917 | 78,450 |
| G | 78,663 | 22,514 | 0 | 22,881 |
| T | 26,521 | 82,881 | 24,179 | 0 |
Supporting Figure 3.
