# Supplementary material for: Comparative transcriptomics and comprehensive marker resource development in mulberry
Source: BMC Genomics. 2016 Feb 4;17:98. doi: 10.1186/s12864-016-2417-8 (PMC4743097; doi:10.1186/s12864-016-2417-8)
Supplement: Additional file 5: — Supporting Figure 4. InDel distribution in mulberry transcriptomes. InDel length distribution in M. laevigata/M. notabilis (A); M. laevigata/M. serrata (B); M. serrata/M. notabilis (C). The x-axis shows InDel length in bp (deletions highlighted with orange line and insertions highlighted by red line) and the y-axis shows the corresponding number of InDels (PPTX 55 kb) [file 12864_2016_2417_MOESM5_ESM.pptx]

## Slide 1
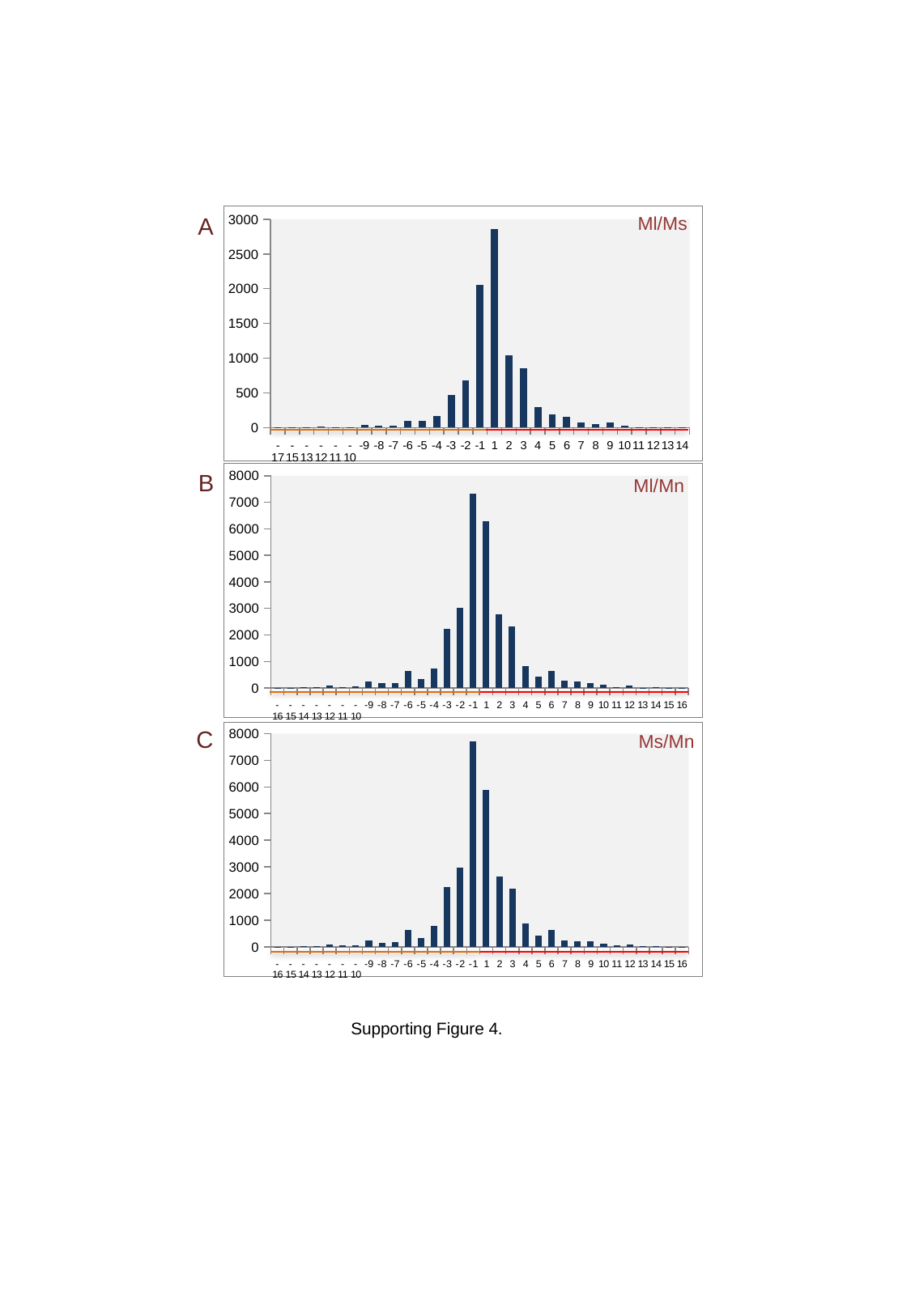

A
### Chart
| Category | Ml/Ms |
|---|---|
| -17.0 | 1.0 |
| -15.0 | 2.0 |
| -13.0 | 2.0 |
| -12.0 | 20.0 |
| -11.0 | 10.0 |
| -10.0 | 8.0 |
| -9.0 | 34.0 |
| -8.0 | 25.0 |
| -7.0 | 32.0 |
| -6.0 | 96.0 |
| -5.0 | 98.0 |
| -4.0 | 166.0 |
| -3.0 | 475.0 |
| -2.0 | 676.0 |
| -1.0 | 2058.0 |
| 1.0 | 2859.0 |
| 2.0 | 1036.0 |
| 3.0 | 851.0 |
| 4.0 | 292.0 |
| 5.0 | 186.0 |
| 6.0 | 161.0 |
| 7.0 | 74.0 |
| 8.0 | 49.0 |
| 9.0 | 74.0 |
| 10.0 | 23.0 |
| 11.0 | 2.0 |
| 12.0 | 6.0 |
| 13.0 | 1.0 |
| 14.0 | 1.0 |Ml/Ms
B
### Chart
| Category | Ml/Mn |
|---|---|
| -16.0 | 3.0 |
| -15.0 | 13.0 |
| -14.0 | 26.0 |
| -13.0 | 24.0 |
| -12.0 | 105.0 |
| -11.0 | 41.0 |
| -10.0 | 68.0 |
| -9.0 | 235.0 |
| -8.0 | 183.0 |
| -7.0 | 181.0 |
| -6.0 | 637.0 |
| -5.0 | 339.0 |
| -4.0 | 743.0 |
| -3.0 | 2232.0 |
| -2.0 | 3013.0 |
| -1.0 | 7324.0 |
| 1.0 | 6292.0 |
| 2.0 | 2776.0 |
| 3.0 | 2322.0 |
| 4.0 | 837.0 |
| 5.0 | 424.0 |
| 6.0 | 629.0 |
| 7.0 | 269.0 |
| 8.0 | 236.0 |
| 9.0 | 197.0 |
| 10.0 | 117.0 |
| 11.0 | 39.0 |
| 12.0 | 86.0 |
| 13.0 | 17.0 |
| 14.0 | 22.0 |
| 15.0 | 12.0 |
| 16.0 | 3.0 |Ml/Mn
C
### Chart
| Category | Ms/Mn |
|---|---|
| -16.0 | 8.0 |
| -15.0 | 12.0 |
| -14.0 | 18.0 |
| -13.0 | 26.0 |
| -12.0 | 82.0 |
| -11.0 | 47.0 |
| -10.0 | 70.0 |
| -9.0 | 241.0 |
| -8.0 | 158.0 |
| -7.0 | 191.0 |
| -6.0 | 629.0 |
| -5.0 | 336.0 |
| -4.0 | 774.0 |
| -3.0 | 2252.0 |
| -2.0 | 2984.0 |
| -1.0 | 7712.0 |
| 1.0 | 5881.0 |
| 2.0 | 2651.0 |
| 3.0 | 2174.0 |
| 4.0 | 867.0 |
| 5.0 | 417.0 |
| 6.0 | 622.0 |
| 7.0 | 234.0 |
| 8.0 | 211.0 |
| 9.0 | 207.0 |
| 10.0 | 111.0 |
| 11.0 | 47.0 |
| 12.0 | 93.0 |
| 13.0 | 23.0 |
| 14.0 | 22.0 |
| 15.0 | 9.0 |
| 16.0 | 3.0 |Ms/Mn
Supporting Figure 4.
