# Supplementary material for: Comparative transcriptomics and comprehensive marker resource development in mulberry
Source: BMC Genomics. 2016 Feb 4;17:98. doi: 10.1186/s12864-016-2417-8 (PMC4743097; doi:10.1186/s12864-016-2417-8)
Supplement: Additional file 7: — Supporting Table 2. List of primers used for Quantitative Real Time PCR (PPTX 53 kb) [file 12864_2016_2417_MOESM7_ESM.pptx]

## Slide 1
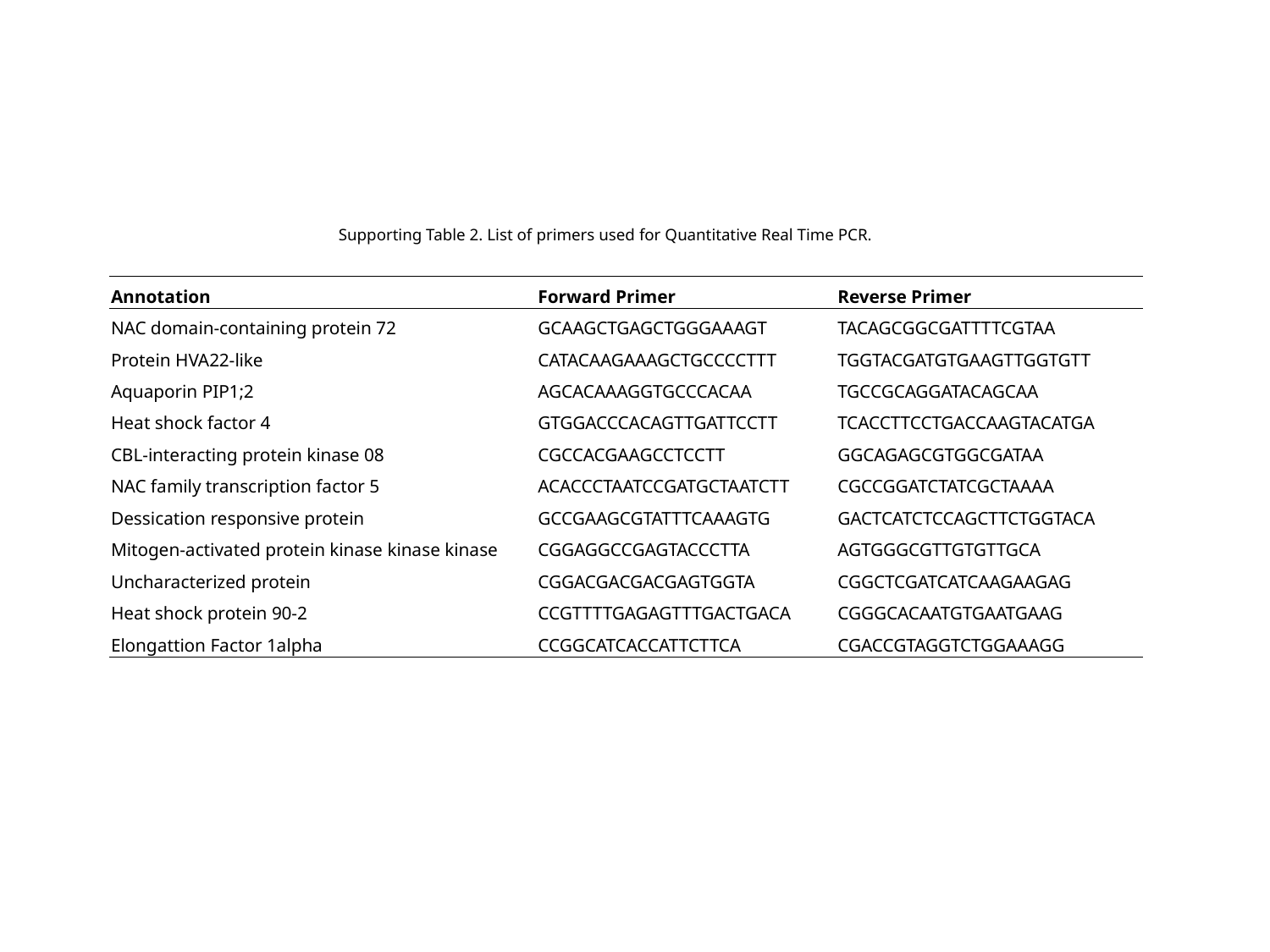

Supporting Table 2. List of primers used for Quantitative Real Time PCR.
| Annotation | Forward Primer | Reverse Primer |
| --- | --- | --- |
| NAC domain-containing protein 72 | GCAAGCTGAGCTGGGAAAGT | TACAGCGGCGATTTTCGTAA |
| Protein HVA22-like | CATACAAGAAAGCTGCCCCTTT | TGGTACGATGTGAAGTTGGTGTT |
| Aquaporin PIP1;2 | AGCACAAAGGTGCCCACAA | TGCCGCAGGATACAGCAA |
| Heat shock factor 4 | GTGGACCCACAGTTGATTCCTT | TCACCTTCCTGACCAAGTACATGA |
| CBL-interacting protein kinase 08 | CGCCACGAAGCCTCCTT | GGCAGAGCGTGGCGATAA |
| NAC family transcription factor 5 | ACACCCTAATCCGATGCTAATCTT | CGCCGGATCTATCGCTAAAA |
| Dessication responsive protein | GCCGAAGCGTATTTCAAAGTG | GACTCATCTCCAGCTTCTGGTACA |
| Mitogen-activated protein kinase kinase kinase | CGGAGGCCGAGTACCCTTA | AGTGGGCGTTGTGTTGCA |
| Uncharacterized protein | CGGACGACGACGAGTGGTA | CGGCTCGATCATCAAGAAGAG |
| Heat shock protein 90-2 | CCGTTTTGAGAGTTTGACTGACA | CGGGCACAATGTGAATGAAG |
| Elongattion Factor 1alpha | CCGGCATCACCATTCTTCA | CGACCGTAGGTCTGGAAAGG |
